# Semi-elemental diet is effective in managing high output ileostomy; a case report 

**Published:** 2019

**Authors:** Suhaib JS Ahmad, Asad Khan, Ravi Madhotra, Aristomenis K. Exadaktylos, Maria Elena Milioto, George Macfaul, Kamran Rostami

**Affiliations:** 1 *School of Medicine, University of Buckingham, Buckingham, UK; *; 2 *Department of Gastroenterology, Milton Keynes University Hospital, Milton Keynes, UK *; 3 *Department of Gastroenterology Palmerston North Hospital, New Zealand *; 4 *Department of Emergency Medicine, Inselspital, University Hospital of Bern, Bern, Switzerland *

**Keywords:** Elemental diet, Short bowel, Ulcerative colitis, Ileostomy, High-output stoma

## Abstract

A notable proportion of surgically created stomas develop high output. Ongoing monitoring and treatment of hight stoma output is imperative to avoid risk of complications. Prevailing management guidelines focus mainly on supportive measures and medications that alter bowel motility. However, some patients fail to respond to these measures, leaving few substitutes. This report documents the use of semi-elemental diet in the management of a high-output ileostomy case. A 58-year-old patient underwent multiple bowel resections that resulted in a small intestine measuring 90 cm, with an end ileostomy being performed. He was on home parenteral nutrition (HPN) for over 9 years and was admitted to the hospital with an episode of sepsis from an infected line. One day prior to the hospital admission, the stoma was producing 7.2 litres/day. The Patient was advised to start Vital 1.5 10-15/day (2.5-3 litres/day) exclusively, in addition to his 1.5 litres of IV fluid, based on the nutritional requirement as calculated by a dietitian. Following the introduction of the semi-elemental diet, the ileostomy output dropped swiftly to 2 litres/day, 9 days post admission, and the BMI remained stable. This report suggests a possible role for semi-elemental diet in the management of ileostomies with short bowel syndrome. Based on our previous experience and this case, elemental or semi-elemental diet may both be used as a mono-therapy, in patients with high ileostomy output, even in cases with small bowel length as short as 90cm.

## Introduction

 In the United Kingdom, approximately 102,000 people live with a stoma, with an estimated 21,000 stomas formed each year (1, 2). High-output stomas (>1500ml for two consecutive days) are poorly addressed by clinicians and are not well identified. An estimated 16% of patients with stomas will suffer from a high output, of whom 7% will require ongoing treatment (3). Patients and clinicians should be made aware of the warning signs of persistent volumes above 1,000 ml/day. Clinicians should also note that patients may just report the secondary effects of high output such as frequent stoma bag emptying, leakage, dizziness and malaise (4, 5). Causes of high output stoma include short bowel, intra-abdominal sepsis and medications (Table 1) (5). Patients with protracted high output are at higher risk of dehydration, acute kidney injury and malnutrition (6). Thus, adequate monitoring of electrolytes, micronutrients and coordination from a multidisciplinary team is indispensable in preventing long-term negative complications and readmissions. Current treatment guidelines for high-output stoma involve mainly fluids restriction, medications like ppi, codeine and loperomide, dietary modifications and patient education (5). Current measures are effective in most patients. However, some patients fail to respond, increasing the need for alternatives.

**Table 1:** Causes of high-output stoma

Causes of high-output stoma
Intra-abdominal sepsis
Enteritis
Intermittent mechanical obstruction
Crohn's disease
Short bowel
Paralytic ileus
Prokinetic medications
Malabsorption disorders
Withdrawal from steroids

**Table 2:** Blood results on admission

Test	Value(Admission)	Value(Discharge)	Units	Normal Range
Sodium	132	138	mmol/L	133-146
Potassium	3.5	4.7	mmol/L	3.5-5.3
Phosphate	0.6	1.2	mmol/L	0.8-1.5
Magnesium	0.6	0.8	mmol/L	0.7-1
WBC	6.3	9.6	10*9/L	3.7-11.1
Neutrophils	5.2	8.1	10*9/L	1.7-7.5
Hb	103	134	g/L	130-170
PLT	267	374	10*9/L	150-450
Urea	5.3	10.3	mmol/L	2.5-7.8
C-reactive protein	103	<2.0	mg/L	0-6
Creatinine	123	84	µmol/l	55-105

The provision of elemental diets (ED) has been part of the primary and adjunctive management of gastrointestinal disorders. Recently, EDs have also been shown to be an effective agent in reducing stoma output (7). The ED consists of essential and non-essential amino acids, fat and sugar. Frequently, water-soluble vitamins, fat-soluble vitamins and electrolytes are added (8). EDs can be given orally or through nasogastric tube if the patient is intolerant to fluids. A variety of uses of ED has been based on the premise that they are more efficiently absorbed, less allergenic, better tolerated in patients with malabsorptive disorders and reduce exocrine pancreatic secretions in patients with pancreatitis (9). The objective of this case report study is to update the literature with a high-output ileostomy case, that has responded following the introduction of semi-elemental diet. 

## Case Report

A 58-year-old Caucasian male, with a 10-year history of ulcerative colitis, almost one year After the diagnosis, had developed anal fistulas, perianal abscesses and villous adenoma. However, multiple stool samples and colonoscopic biopsies had ruled out co-existent superinfections. Consecutively, the patient had a proctocolectomy with ileal pouch-anal anastomosis, followed by small bowel resections due to adhesions complications. This resulted in a 90 cm small intestine, with a permanent end ileostomy being performed. After his multiple operations resulting in a short bowel, he was treated parenterally via a central venous catheter (Hickman line). Since then, the patient has had multiple central venous catheter-associated infections and has had his line changed several times. The patient was under the care of St Mark’s hospital and had no significant family or social history. He was admitted to the Emergency Department, at Milton Keynes University Hospital, presenting with fever and rigors. Blood tests were manifested by anaemia and raised inflammatory markers. The patient was put on several anti-motility and anti-secretory medications (Loperamide, Codeine phosphate and Omeprazole) to reduce the stoma output. 

The patient had no other symptoms aside from feeling generally unwell, fever and rigors. Initial assessment revealed a heart rate of 108, a respiration rate of 24, an SpO2 of 93%, a temperature of 38.9° C, a blood pressure of 154/94 mmHg and a GCS score of 15/15. With the exception of abdominal surgical scars and a Hickman line over the left side of chest, general physical examination was unremarkable including normal heart sounds with no added sounds. The Hickman line was not loose or disconnected. The line was not blocked. The patient’s stoma was situated in the right iliac fossa. There were no signs of swelling, bruising, pain, bleeding, redness, or oozing around the exit site. There were no signs of parastomal hernia, prolapse, retraction, infarction, bleeding or erosions. However, the patient complained of tenderness around the stoma site. Further inspection also revealed a high stoma output of 7.2 litres per day, one day prior to the admission; which might have contributed to the electrolyte and water imbalance (Table 2). Chest x-ray and ECG were unremarkable. Urinalysis revealed hematuria and proteinuria. Multiple sets of peripheral and central blood cultures were taken. Growth, detected in blood cultures, resembled Staphylococcus epidermis. Since there were no other signs of line involvement, the current Hickman line was kept in-situ and the patient was treated with antibiotics and monitored closely. The patient was managed with IV fluids, analgesia, antiemetics and with several IV antibiotics (Flucloxacillin, Teicoplanin, Gentamicin and Tazocin) following the advie of microbiologist. Over the course of Vancomycin therapy, the patient had no further temperature spikes and the C-reactive protein value returned to the normal limits. 

The patient was then referred to a dietitian to be commenced on semi-elemental diet (Vital 1.5). The BMI was measured at 24.5kg/m^2^ with a Must score of 0. His estimated nutrition requirements are shown in Table 3. The hospital protocol for high stoma output is 500ml of oral fluids and 1litre of St Mark’s solution. Initially, the patient was started on 8 bottles per day of Vital 1.5 (2400kcal/18.0% Protein, 49.0% Carbohydrate, 33.0% Fat) in addition to the St Mark’s solution. Due to the large stoma output, he was on large amount of fluids that gradually reduced following an improvement in the stoma output by semi-elemental diet contribution. There were no changes in medications that could have accounted to the stoma output drop. 

**Table 3:** Daily Nutrition requirements

Daily Nutrition requirements	
Energy	2500-2947 kcal
Protein	89-131 g
Fluids	2940 ml

-8.3 bottles of vital 1.5kcal would meet lower end of the nutrition requirement.

* Vital 1.5kcal can be presented as 1000 ml ready to hang containers and 200 ml recloseable plastic bottles.

**Figure 1:**
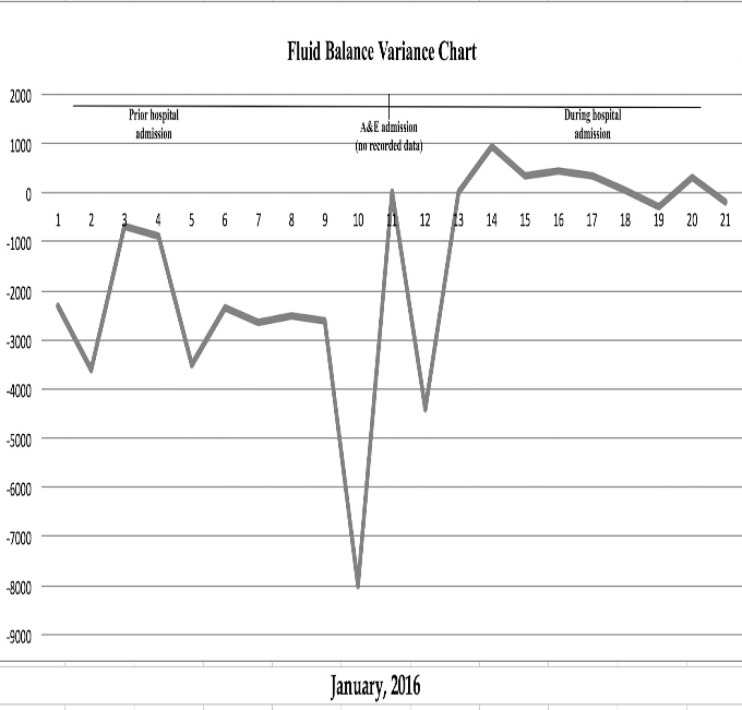
Fluid Input vs Output Fluid balance Chart

Three days post-admission, the dose of Vital 1.5 was increased to 10-15 bottles (3000kcal-4500 kcal/18.0% Protein, 49.0% Carbohydrate, 33.0% Fat) per day in addition to his fluid intake that was restricted to 3 litre of overall and 2 litres of IV fluid. Following a further improvement in the stoma output, the patient was advised to stop the Codeine phosphate, Loperamide, the St Mark’s solution and further reducing/adjusting his fluid intake. However, the patient only agreed to stop the St Mark’s solution. 1 litre of Plasma-Lyte was to be administered in case of dehydration. The introduction of semi-elemental diet (Vital 1.5kcal) has led the stoma output to drop swiftly to 2 litres/day, 9 days post admission (Figure 1).

**Figure 2:**
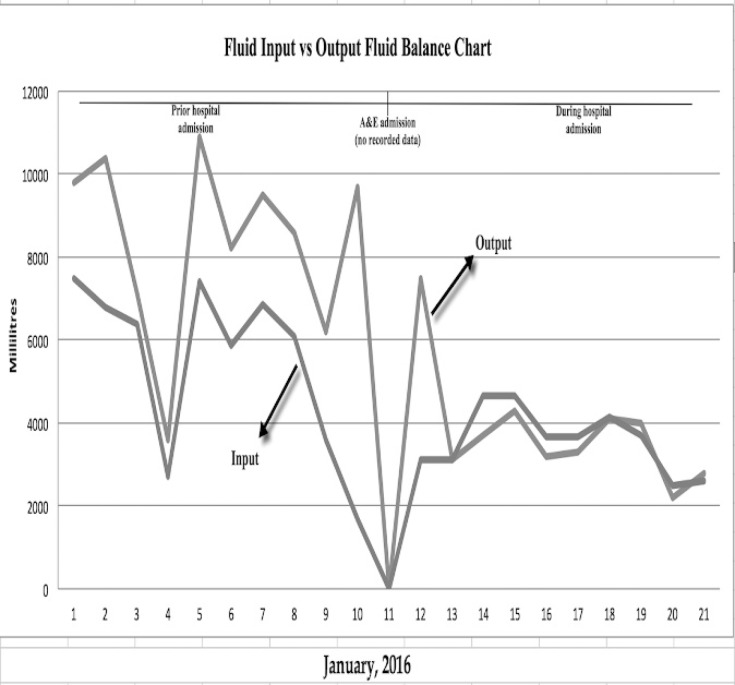
Fluid Balance Variance Chart

Fluid balance charts in hospital inpatients have been used to monitor the fluid balance (Figure 2). The patient maintained a stable BMI and the electrolytes returned to within the normal limits (Table 2). Prior to discharge, a full vitamin screen was performed, to act as a baseline for future reference. Furthermore, the patient was told to continue monitoring his weight, fluid balance and stoma output. Consent was obtained from the patient for publication of this case report. 

## Discussion

The gut handles approximately 8-10 Litres of fluid daily, most of which is absorbed by the jejunum and the ileum. An estimated 1.5 Litres reaches the colon, of which 100 ml is excreted (10,11). Where there is an ileostomy, the stoma output depends immensely on the length of the small bowel. In the case of extensive intestinal resections, the absorptive surface, which is responsible for the absorption of water, electrolyte and nutrients, is significantly reduced (12). This has led the patient, described in this case, to have a high stoma output. Studies have shown that resecting up to half of the small intestine is well tolerated among patients (13). Following extensive resections, the gut undergoes physiological adaptation to restore gut absorption to the pre-insult state. Adaptations include increasing the surface area for absorption and slowing down the gastrointestinal transit. These patients experience a gradual reduction in nutritional requirements over time. Short bowel syndrome develops when the residual small intestine length is less than two meters (14). 

A large proportion of patients with short bowel syndrome, have persistent high stoma output and require ongoing parental nutritional support. The patient described in this manuscript, underwent extensive small bowel resections that resulted in short bowel syndrome (90 cm small intestine) (15). The patient was on parental nutritional support and reported a consistent high stoma output despite fluid restrictions and consuming glucose-saline solutions collectively with regular doses of anti-motility and anti-secretory agents. Semi-elemental diet (Vital 1.5k) was a very effective agent in reducing the stoma output. With such a high stoma output, malabsorption would easily occur. An elemental or polymeric diet may improve the absorption profile as supported by several studies (9). For instance Christie DL *et al*., demonstrated evidence that a dilute ED, administered by continuous intra-gastric infusion in large volumes, may be very useful in treating patients with short bowel syndrome (16).

The mechanism of action of semi-elemental diet might be multifactorial. It seems possible that the reduction in the workload of digestion and absorption by semi-elemental diet and in peristalsis and digestive tract secretions may play a role in reducing the residual faeces; thus decreasing the stoma output. Furthermore, increased gut permeability has been shown to be an important factor in the pathogenesis of inflammatory bowel disease and ED have been shown to decrease intestinal permeability; thus decreasing fluid loss (17). It seems possible that many antigens that can induce inflammation in the bowel can be avoided through the use of ED and semi-elemental diet. Semi-elemental diet might also reduce the commensal gut bacteria that play a role in bowel inflammation. 

Despite clinical practice guidelines and emerging evidence suggest that dietary changes would ameliorate the gastrointestial symptoms, many patients continue to receive minimal dietary instruction. Both elemental and semi-elemental diets have a crucial role in managing several gastrointestinal disorders as reviewed previously (7). 

***Limitation of the study***

This Case report has no information on the long-term nutritional effect of semi-elemental diet. Causality cannot be deduced from this single uncontrolled observation. An association between the use of semi-elemental diet and reduced stoma output does not imply a cause-effect relationship. The reduction in stoma output following the introduction of semi-elemental diet could be a mere coincidence. However, all descriptive studies share this limitation. As a result, findings from a case report cannot be generalized unless a cause-effect relationship from a representative population is established. Case reports aim to contribute to a change in the clinical practice. Numerous discoveries and major advancements in medicine started with a case report. 

## Conclusion

Patients with high output ileostomies due to short bowel syndrome should each be managed individually. They are all different in diagnosis, bowel length and characteristics. ED seem to be a safe and effective mean for managing patients with high output ileostomy. ED also enable clinicians to discontinue the use of parenteral alimentation sooner and provide adequate nutrition in patients with high output stoma, who might have failed any other therapeutic formula. Furthermore, a plethora of evidence suggests that elemental and semi-elemental diets have a significant role in treating and providing nutritional support in patients with gastrointestinal diseases.

## Conflict of interests

The authors declare that they have no conflict of interest.
